# Propofol Protects Hippocampal Neurons from Hypoxia-Reoxygenation Injury by Decreasing Calcineurin-Induced Calcium Overload and Activating YAP Signaling

**DOI:** 10.1155/2018/1725191

**Published:** 2018-06-26

**Authors:** Xiaojun Li, Li Yao, Qianlei Liang, Hangyin Qu, Hui Cai

**Affiliations:** ^1^The Second Department of Thoracic Surgery, The First Affiliated Hospital of Xi'an Jiaotong University, Xi'an, Shaanxi 710061, China; ^2^Department of Vascular Surgery, The First Affiliated Hospital of Xi'an Jiaotong University, Xi'an, Shaanxi 710061, China; ^3^The Second Department of Neurosurgery, China-Japan Union Hospital of Jilin University, Changchun 130033, China; ^4^Shaanxi University of Chinese Medicine, Xianyang, Shaanxi 712000, China

## Abstract

**Objectives:**

Propofol is a popular anesthetic drug that is neuroprotective. However, the mechanisms of propofol for hippocampal neuroprotection remain elusive. This study is aimed at investigating the neuroprotective effect and mechanism of propofol in hippocampal neurons exposed to ischemia-reperfusion (I/R) injury.

**Methods:**

Hypoxia-reoxygenated (H/R) HT-22 cells were used to mimic I/R injury of the hippocampus in vitro. An MTT assay was used to determine cell viability. Cell apoptosis was detected by a TUNEL assay and a flow cytometry cell apoptosis assay. Expression levels of proteins were measured by Western blotting. Intracellular calcium was assessed by Fura-2/AM staining. Flow cytometry was used to determine the mitochondrial membrane potential (MMP). Coimmunoprecipitation was used to evaluate the stability of the FKBP-RyR complex. Calcineurin enzymatic activity was measured with a colorimetric method. YAP nuclear translocation was tested by immunofluorescence staining.

**Results:**

H/R induced HT-22 cell viability depression, and apoptosis was reversed by propofol treatment. Propofol could alleviate H/R-induced intracellular calcium accumulation and MMP loss by inhibiting calcineurin activity and FKBP12.6-RyR disassociation in a concentration-dependent manner. In addition, YAP expression was crucial for propofol to protect HT-22 cell apoptosis from H/R injury. Propofol could activate YAP through dephosphorylation. Activated YAP stimulated the transcription of the Bcl2 gene, which promotes cellular survival. Our data also demonstrated that propofol activated YAP through the RhoA-Lats1 pathway without large G proteins or MST involvement. In addition, we showed that there was no interaction between calcineurin signaling and YAP activation in HT-22 cells.

**Conclusions:**

Propofol protected hippocampal neurons from I/R injury through two independent signaling pathways, including the calcineurin/FKBP12.6-RyR/calcium overload pathway and the RhoA/Lats1/YAP/Bcl-2 pathway.

## 1. Introduction

Ischemic stroke has become one of the leading causes of morbidity and mortality worldwide [1]. To treat ischemic injury, reestablishment of blood supply for the ischemic region is the most effective approach [2]. However, cerebral ischemia-reperfusion (I/R) injury after sudden recovery of blood supply, causing dysfunction of neurons, glia cells, and cerebral blood vessels, still threatens the survival of stroke patients [3]. Previous studies indicated that neuronal apoptosis was the associated mechanism of I/R injury, and the pyramidal neurons were found to be the most vulnerable neurocytes to I/R injury-induced apoptosis [4]. Over recent decades, numerous studies were conducted to prevent hippocampal neurons from I/R injury. Among them, anesthetic drugs have been suggested to have neuroprotective effects on cerebral I/R injury via inhibiting cell apoptosis [5–7].

Propofol, also known as 2,6-disopropylphenol, has been a widely used intravenous short-acting anesthetic agent since the late 1980s. It was reported that, except for its benefits as an anesthetic agent, propofol also exerts many nonanesthetic effects, including immunomodulatory effects, analgesia effects, anxiolytic effects, and neuroprotective properties [8]. Previous studies indicated that propofol could reduce hypoxia/reoxygenation- (H/R-) induced cell apoptosis of myocytes, epithelial cells, and neurons [9, 10]. Several mechanisms were mentioned, such as mitochondrial dysfunction, apoptosis-inducing factor translocation, and the m-TOR pathway [7, 11]. Recently, propofol was also shown to inhibit rat hippocampal neuronal apoptosis by depressing calcium overload [6]. Of note, propofol could regulate multiple intracellular signaling pathways [8]. The mechanisms involved in the propofol's neuroprotective role in hippocampal neurons need more exploration.

YAP (Yes-associated protein) is a transcriptional coactivator that is negatively regulated by the Hippo pathway, which originally was identified for the function in the regulation of organ development and size [12]. Subsequent studies verified the effects of YAP in neuronal proliferation, survival, differentiation, and neurogenesis in both the central and peripheral nervous systems [13–17]. Meanwhile, the intracellular signaling that regulates YAP activation was widely discussed [18–21]. However, the extracellular regulators and detailed mechanisms of YAP signaling in hippocampal neurons are essentially unknown. At present, activation of YAP is best known to be regulated by multiple phosphokinases and phosphatases [22, 23]. Since propofol could regulate activation of phosphokinases and phosphatases [5, 24], we hypothesized that propofol would have neuroprotective effects in I/R injury, perhaps through activating YAP signaling.

In this study, we used hypoxia-reoxygenated hippocampal neurons in vitro to mimic I/R injury of the hippocampus and then aimed to confirm that propofol could prevent hippocampal neurons from hypoxia/reoxygenation- (H/R-) induced apoptosis by decreasing calcineurin-induced calcium overload. Furthermore, the roles and mechanism of YAP signaling in propofol alleviating H/R-induced hippocampal neuronal apoptosis were also explored. Meanwhile, we aimed to clarify whether there is cross-talk between the calcineurin-calcium pathway and YAP signaling in hippocampal neurons.

## 2. Materials and Methods

### 2.1. Reagents

Propofol was purchased from Sigma-Aldrich (St. Louis, MO, USA). The following inhibitors were used in this study: pertussis toxin (PTX; Invitrogen, Grand Island, NY, USA), Y27632 (BioVision, Milpitas, CA, USA), C3-exoenzyme (Cytoskeleton, Denver, CO, USA), and cyclosporine A (Selleckchem, Houston, TX, USA). YAP, phospho-YAP (Ser127), phosphoLats1 (Ser909), and phospho-MST1 (Thr183)/MST2 (Thr180) antibodies were purchased from Cell Signaling (Boston, MA, USA). GAPDH, Bax, caspase-3, caspase-9, survivin, and Bcl2 antibodies were purchased from Santa Cruz Biotechnology (Santa Cruz, CA, USA). Alexa Fluor secondary antibodies were purchased from Life Technologies, Grand Island, NY, USA. The dominant negatives (dn) of large and small G protein constructs were from UMR cDNA Resource Center (Rolla, MO, USA).

### 2.2. Cell Culture and Treatment

HT-22 cells, which were derived from immortalized mouse hippocampal neuron cultures, were provided by ATCC. Cells were cultured with Dulbecco's modified Eagle's medium (DMEM, Gibco, Thermo Fisher Scientific, Shanghai, China) supplemented with 10% fetal bovine serum (FBS, Gibco, Thermo Fisher Scientific, Shanghai, China) and an antibiotics mix (HyClone, GE Healthcare, Pittsburgh, PA, USA) in a humidified cell incubator with 5% CO_2_ and 95% fresh air at 37°C.

### 2.3. Hypoxia-Reoxygenation Protocol

The protocol performed was in accordance with previous studies [25]. Briefly, the culture plates were transferred to a humidified hypoxia-controlled incubator chamber (1% O_2_, 5% N_2_, 94% CO_2_) at 37°C for 6 hours. Then, these cells were exposed to reoxygenation in a humidified incubator providing atmosphere containing 5% CO_2_ and 95% fresh air at 37°C for 6 hours.

### 2.4. Cell Viability Assay

An MTT assay was used in this study to assess the viability of the neurons. Briefly, after treatment, cells were seeded in the wells of 96-well culture plates. MTT was added to the cells at a final concentration of 0.5 mg/ml and incubated for 4 hours at 37°C. DMSO was added to dissolve the formed formazan crystals. A microplate reader (Bio-Rad, Hercules, California, USA) was used to determine the absorbance values at 490 nm.

### 2.5. Cell Apoptosis Assessment

Cell apoptosis was evaluated by terminal deoxynucleotidyl transferase- (TdT-) mediated dUTP nick end labeling (TUNEL) assay in this study. After treatment, cells were fixed with paraformaldehyde (4%) at room temperature for 30 minutes. Then, Triton X-100 (0.1%, Solarbio) was used to permeabilize the cells at room temperature for 30 minutes. A TUNEL assay kit (Roche, Indianapolis, IN, USA) was used to visualize the apoptotic cells. All procedures performed were in accordance with the manufacturer's instructions. A fluorescence microscope was used to observe the TUNEL-positive cells.

### 2.6. Flow Cytometry Cell Apoptosis Assay

For the cell apoptosis assay, cells were harvested after designated treatments, washed twice with PBS, and resuspended in binding buffer (BioLegend, San Diego, CA, USA). Cells were incubated with PI and FITC-Annexin V (BioLegend, San Diego, CA, USA) for 30 min, and the percentage of apoptotic cells was analyzed by flow cytometry. Three independent repeated experiments were performed.

### 2.7. Coimmunoprecipitation

After treatment, lysis buffer (0.1 Triton X-100, 100 mmol/l NaCl, 50 mmol/l Tris HCl, 10 mmol/l EDTA, 10 mmol/l NaF, 10% glycerol, 0.5 mmol/l phenylmethylsulfonyl fluoride, 1 mmol/l dithiothreitol, and 10 mmol/l sodium pyrophosphate) was used to lyse the cells. After centrifugation at 12000*g* for 5 minutes, protein-G agarose (Beyotime, Shanghai, China) was incubated with a specific antibody against RyR (Cell Signaling, Boston, MA, USA) in the supernatants and rotated for 12 hours. The specific antibody for FKBP12.6 (Cell Signaling, Boston, MA, USA) was used for immunoblotting. Recruitments of molecules were calculated based on density detection.

### 2.8. Intracellular Calcium Assessment

The calcium indicator, Fura-2/AM (Invitrogen, Grand Island, NY, USA), was used in this study. After treatment, cells were incubated with Fura-2/AM at 10 *μ*mol/l at room temperature for 30 minutes. After washing, a fluorescent inverse microscope was used to observe the cells at 510 nm after excited at 340 nm and the images were analyzed by Zeiss Physiology software (v3.2, Zeiss). Mean fluorescent intensity (MFI) was used to analyze intracellular calcium concentrations.

### 2.9. Mitochondrial Membrane Potential (MMP) Determination

MMP was determined by detection of the MMP indicator, rhodamine 123, by flow cytometry in accordance with the descriptions from previous studies. After treatment, cells were washed and incubated with rhodamine 123 solution (Beyotime, Shanghai, China) at a final concentration of 1 *μ*mol/l at 37°C in a dark chamber for 30 minutes. A FACS cytometer (BD Biosciences, CA, USA) was used to detect the fluorescent signal of rhodamine 123 at 529 nm.

### 2.10. Plasmids and shRNA Transfection

6-Well plates were seeded with 5 × 104 cells/well in 2 ml media 24 hr before transfection; cells were 80%–90% confluent. Cells were transfected with shRNA (100 pmol/well) or plasmid DNA (4 *μ*g/well) using Lipofectamine 2000 Reagent (Life Technologies, Grand Island, NY, USA) according to the manufacturer's instructions. After 48 hr of transfection, cells were used for further experiments. All shRNAs were purchased from Santa Cruz Biotechnology (Santa Cruz, CA, USA).

### 2.11. Immunofluorescence Staining

Cells were seeded in chamber slides. After treatment, cells were fixed with 4% paraformaldehyde-PBS for 15 min. Following blocking in 5% goat serum with 0.3% Triton X-100 in PBS for 60 min, cells were incubated with YAP primary antibody (1 : 100 dilution) overnight at 4°C. After three washes with PBS, cells were incubated with Alexa Fluor 488- or 555-conjugated secondary antibodies (Invitrogen, 1 : 500 dilution) for 2 hr at room temperature. Slides were then washed three times and mounted. Immunofluorescence was detected using a QImaging Retiga 2000R camera (Surrey, BC, Canada) at 40x magnification. For frozen tissues, 5 *μ*m sections were prepared and subjected to immunostaining as described.

### 2.12. Western Blotting

After treatment, RIPA cell lysis buffer (Santa Cruz, CA, USA) was used to lyse the cells. Total protein was extracted with a Protein Extraction Kit (Beyotime, Shanghai, China), and nuclear and cytosolic protein was extracted using the Nuclear-Cytosol Extraction Kit (TDY, Biotech Co. Ltd., Beijing) according to the manufacturer's instructions. After the protein samples were subjected to SDS-PAGE, the separated proteins were transferred to polyvinylidene difluoride (PVDF) membranes. Nonspecific binding was eliminated by incubation with blocking buffer. Then, specific antibodies against Bax, caspase-3, caspase-9, YAP, pYAP, MST1/2, pMST1/2, Lats1, pLats1, and GAPDH were used to incubate the membranes, which were then incubated with fluorescent secondary antibodies (IRDye800CW-conjugated or IRDye680-conjugated antispecies IgG, LI-COR Biosciences, Lincoln, NE, USA). The fluorescent signals were captured by an Odyssey Infrared Imaging System (LI-COR Biosciences, Lincoln, NE, USA) with both 700 and 800 nm channels. Boxes were manually placed around each band of interest, and the software returned near-infrared fluorescent values of raw intensity with background subtraction (Odyssey 3.0 analytical software, LI-COR Biosciences, Lincoln, NE, USA).

### 2.13. Calcineurin Activity Assay

Enzymatic activity of calcineurin was measured and calculated with a colorimetric method with the total protein extraction from treated cells. A calcineurin Activity Assay Kit (Merck) was used, as per the manufacturer's instructions.

### 2.14. Quantitative Real-Time PCR

After shRNA transfection for 48 hr, cells were washed with cold PBS and collected in the Qiagen RLT lysis buffer (Qiagen, Valencia, CA, USA). RNA was extracted with an RNeasy mini kit (Qiagen, Valencia, CA, USA) and reverse transcribed by M-MLV reverse transcriptase. Quantitative real-time PCR was performed on a Light Cycler 480 (Roche, Indianapolis, IN) with a SYBR Green I Master Mix (Roche, Indianapolis, IN). mRNA abundance was normalized to GAPDH. Negative controls contained no transcripts or reverse transcriptase. RNA from three separate cell pellets per treatment was analyzed. Relative gene expression was calculated using the method provided by Applied Biosystems User Bulletin Number 2 (P/N 4303859B), with nontargeting shRNA-treated cells acting as the control in each data set. Primer pairs used in this study were as follows: GAPDH: F, 50-GAAGGTGAAGGTCGGAGT-30/R, 50-GAAGATGGTGATGGGATTTC-30; survivin: F, 5′-GGACCACCGCATCTCTACAT-3′/R, 5′-GCACTTTGCCAGTTTCC-3′; and Bcl2: F, 5′-TTCTTTGAGTTCGGTGGGGTC-3′/R, 5′-TGCATATTTGTTTGGGGCAGG-3′.

### 2.15. Statistics

Data acquired in this study are presented as mean ± SEM. Data were analyzed by SPSS software (v17.0, SPSS). Differences between groups were evaluated by ANOVA or Student's *t*-tests. *P* values < 0.05 were considered statistically significant.

## 3. Results

### 3.1. Propofol Alleviated Hypoxia-Reoxygenation- (H/R-) Induced Hippocampal Neuronal Viability Depression and Apoptosis

**Figure 1:**
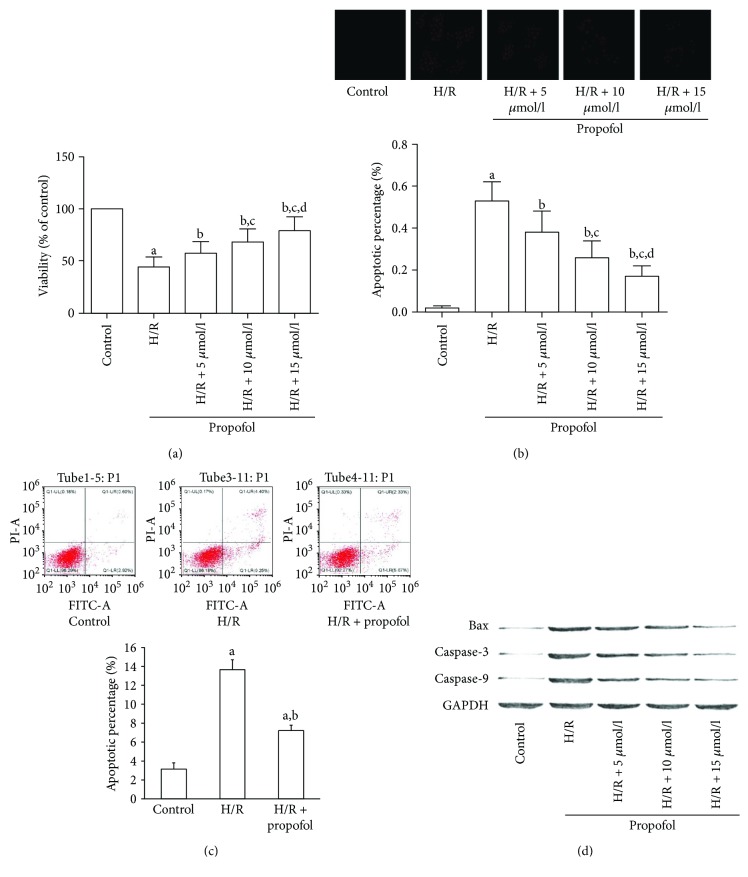
Propofol attenuated hypoxia-reoxygenation, suppressed viability, and induced apoptosis of HT-22 cells. (a, b, and c) HT-22 cells were pretreated without or with different concentrations of propofol for 2 hr prior to stimulation with hypoxia-reoxygenation (H/R). Cell viability was analyzed by MTT (a). Cell apoptosis was tested by TUNEL assay (b) and flow cytometry cell apoptosis assay (c, 15 *μ*mol/l propofol). (d) Expression levels of Bax, caspase-3 (cleaved), and caspase-9 in HT-22 cells with different treatments were analyzed by Western blots. (^a^Differences were significant when compared with “control,” *P* < 0.05. ^b^Differences were significant when compared with “H/R,” *P* < 0.05. ^c^Differences were significant when compared with “H/R + 5 *μ*mol/l,” *P* < 0.05. ^d^Differences were significant when compared with “H/R + 10 *μ*mol/l,” *P* < 0.05.)

MTT was used to assess cell viability. After H/R treatment, the cell viability of hippocampal neurons was significantly inhibited. However, propofol pretreatment dramatically improved the viability of HT-22 cells in a concentration-dependent manner (Figure 1(a)). We further tested the effects of propofol in cell apoptosis induced by H/R. As shown in Figures 1(b) and 1(c), H/R significantly elevated the apoptotic rate of hippocampal neurons by using the TUNEL assay (Figure 1(b)) and flow cytometry cell apoptosis assay (Figure 1(c)). However, apoptosis was suppressed in neurons that received propofol treatment. Consistently, protein expression levels of the apoptosis markers, namely, Bax, caspase-3, and caspase-9, were dramatically elevated in H/R neurons and restored when pretreated with propofol (Figure 1(d)).

### 3.2. Propofol Alleviated H/R-Induced Intracellular Calcium Accumulation and MMP Loss by Inhibiting Calcineurin Activity and FKBP12.6-RyR Disassociation

**Figure 2:**
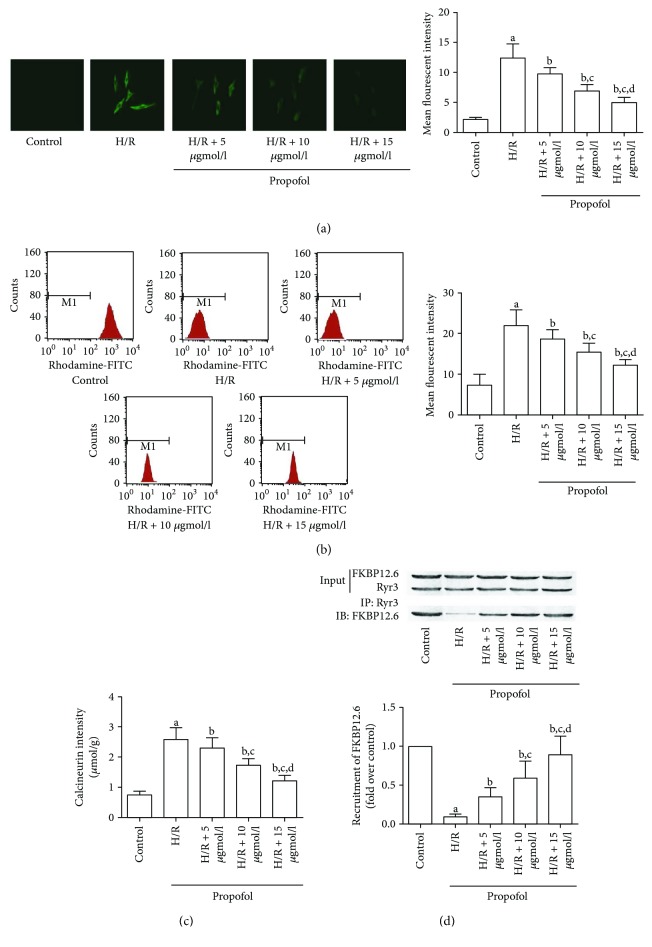
Propofol reversed H/R-induced intracellular calcium accumulation and MMP loss by inhibiting calcineurin activity and FKBP12.6-RyR disassociation. HT-22 cells were pretreated without or with different concentrations of propofol for 2 hr prior to stimulation by H/R. (a) Intracellular calcium was assessed by the calcium indicator, Fura-2/AM. Typical images of Fura-2A/M staining were captured. Columns indicate the measured mean fluorescent intensities of Fura-2A/M staining in HT-22 cells. (b) MMP measurements were detected by flow cytometry. Columns indicated the measured mean fluorescent intensities of rhodamine 123 staining in HT-22 cells. (c) Columns demonstrated the detected enzymatic activity of calcineurin in HT-22 cells. (d) FKBP12.6-RyR disassociation in HT-22 cells was detected by coimmunoprecipitation. RyR3 was immunoprecipitated with FKBP12.6 antibody and immunoblotted with FKBP12.6 antibody. Columns indicate the molecular recruitment of FKBP12.6. (^a^Differences were significant when compared with “control,” *P* < 0.05. ^b^Differences were significant when compared with “H/R,” *P* < 0.05. ^c^Differences were significant when compared with “H/R + 5 *μ*mol/l,” *P* < 0.05. ^d^Differences were significant when compared with “H/R + 10 *μ*mol/l,” *P* < 0.05.)

Since intracellular calcium overload was recognized as an initiator of cell apoptosis [26], we detected the changes of intracellular calcium content in HT-22 cells that suffered from H/R with or without propofol. As shown in Figures 2(a) and 2(b), H/R treatment significantly increased intracellular calcium and impaired MMP. However, in propofol-treated neurons, both calcium accumulation and MMP loss were attenuated in a concentration-dependent manner. We further studied the mechanism of propofol-inhibited H/R-induced intracellular calcium accumulation. As a result, H/R treatment significantly increased the calcineurin activity, which was reversed by propofol in a concentration-dependent manner (Figure 2(c)). Furthermore, the co-IP assay found more FKBP12.6 disassociated from RyR3 after H/R treatment. However, when neurons were treated with propofol, FKBP12.6 molecules were recruited to bind with RyR3 (Figure 2(d)).

### 3.3. Downregulation of YAP Impaired Protective Effects of Propofol in Hippocampal Neuronal Apoptosis Induced by H/R

**Figure 3:**
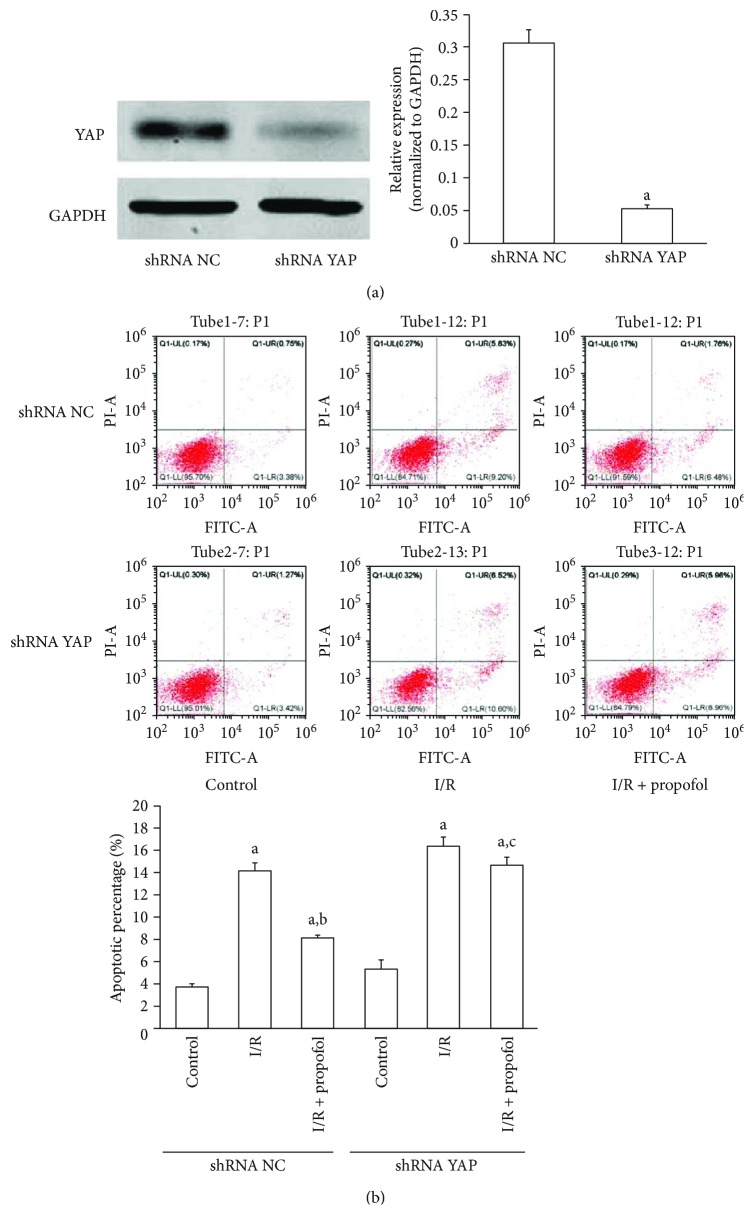
Suppression of YAP impaired protective effects of propofol in HT-22 cell apoptosis induced by H/R. (a) Reduced YAP expression by YAP shRNA in HT-22 cells detected by Western blots. ^a^*P* < 0.001 versus shRNA NC. (b) The effect of downregulation of YAP on cell apoptosis induced by H/R with or without propofol (15 *μ*mol/l) pretreatment in HT-22 cells (conducted 48 hr post shRNA treatment). The results are from three independent experiments. (^a^Differences were significant when compared with “control,” *P* < 0.05. ^b^Differences were significant when compared with “H/R,” *P* < 0.05. ^c^Differences were not significant when compared with “H/R,” *P* > 0.05.)

The potential involvement of YAP in propofol protecting hippocampal neurons from H/R-induced apoptosis was tested. Stable HT-22 cells with low expression of YAP were established using shRNA (Figure 3(a)). As a result, YAP knockdown significantly impaired propofol inhibiting HT-22 cell apoptosis induced by H/R (Figure 3(b)).

### 3.4. Propofol-Induced Dephosphorylation and Nuclear Translocation of YAP in HT-22 Cells

**Figure 4:**
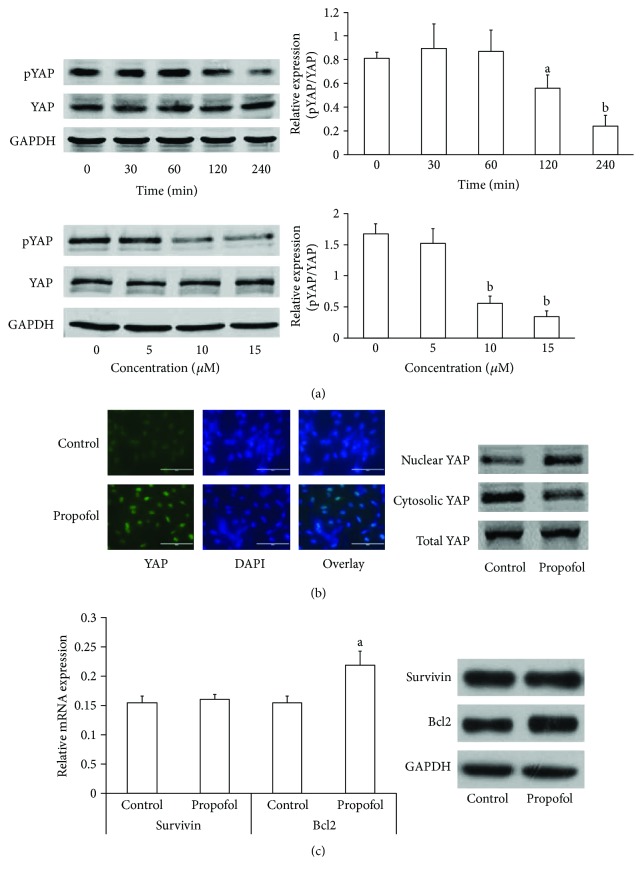
Propofol induced dephosphorylation and nuclear translocation of YAP in HT-22 cells. (a) HT-22 cells were starved for 16 hr, then treated with propofol (15 *μ*mol/l) for different times or with different concentrations of propofol for 4 hr. Western blots were used to analyze the expression of phosphorylated YAP and total YAP. Representative results are shown from three independent experiments. ^a^*P* < 0.01 versus 0 min, ^b^*P* < 0.001 versus 0 min. (b) Propofol-induced (15 *μ*mol/l for 4 hr) YAP nuclear translocation is shown in starved HT-22 cells by immunofluorescence staining and Western blots. Green: YAP; blue: DAPI. (c) Starved HT-22 cells treated with 15 *μ*mol/l propofol for 12 hr, then mRNA and protein levels of survivin and Bcl2 were detected by real-time PCR and Western blot. ^a^*P* < 0.05 versus control.

We tested whether propofol affected the dephosphorylation of YAP (dpYAP) at Ser127 in hippocampal neurons. Propofol induced dpYAP in a dose- and time-dependent manner in HT-22 cells, with the maximal effect at 4 hr and at 15 *μ*M (Figure 4(a)). Concomitantly, propofol also induced YAP nuclear translocation (Figure 4(b)). Activated YAP stimulates the transcription of genes that promote cellular survival, such as survivin and Bcl2 [12, 27, 28]. We further tested the roles of propofol in expression of survivin and Bcl2. As shown in Figure 4(c), propofol significantly increased Bcl2 expression in both mRNA and protein levels. However, there was no change of survivin expression after propofol treatment in HT-22 cells.

### 3.5. Propofol Activated YAP through RhoA-Lats1

**Figure 5:**
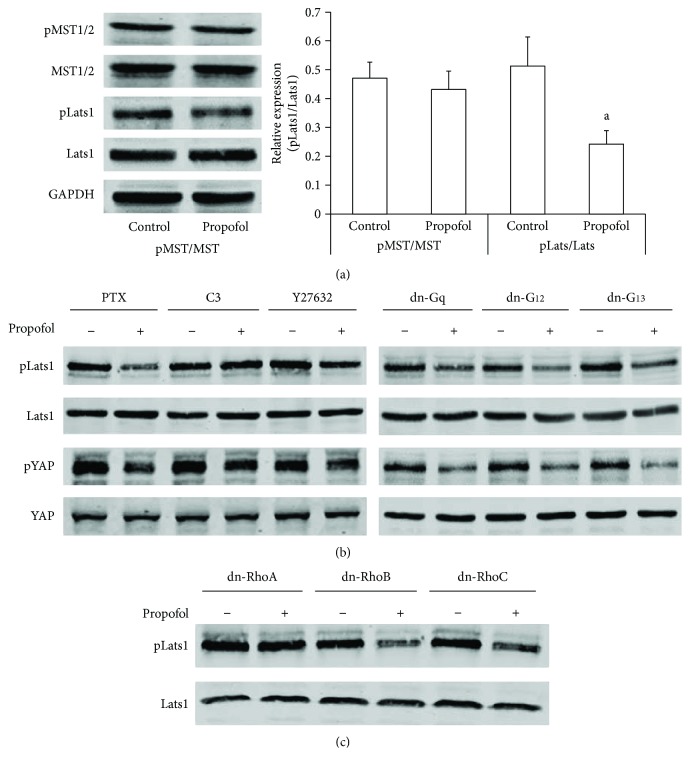
Propofol dephosphorylated YAP through the RhoA-Lats1 signaling pathway. (a) Starved HT-22 cells were treated with propofol (15 *μ*mol/l) for 4 hr, and pMST1/2, total MST1/2, pLats1, and Lats1 were analyzed by Western blot. Representative results are shown from three independent experiments. ^a^*P* < 0.001 versus control. (b) Cells were pretreated with PTX (100 ng/ml, 16 hr), C3 transferase (1 *μ*g/ml for 2 hr), and Y27632 (10 *μ*mol/l for 2 hr) or transfected with different dn plasmids for 48 hr, then starved and treated with propofol (15 *μ*mol/l for 4 hr). Cell lysates were analyzed by Western blot. Representative results are shown. (c) HT-22 cells were transfected with different dn-Rho plasmids, then starved and treated with propofol (15 *μ*mol/l for 4 hr). Expression of pLats1 and Lats1 was analyzed by Western blot.

To determine whether propofol acts through the Hippo pathway core components to regulate YAP phosphorylation, we examined the effect of propofol on phosphorylation of MST1/2 (phospho-MST1 (Thr183)/MST2 (Thr180)) and Lats1 (phospho-LATS1 (Ser909)). We found that propofol had no detectable effect on MST1/2 phosphorylation in HT-22 cells. However, propofol dephosphorylated Lats1 (Figure 5(a)). Since G protein signaling has been proved to regulate Lats activation, we further tested the roles of G proteins in propofol-induced dephosphorylation of Lats1 (dpLats1) and YAP (dpYAP) by specific pharmacological inhibitors and dominant negative (dn) forms of G proteins. As shown in Figure 5(b), pertussis toxin (PTX, inhibitor of Gi protein), dn-Gq, dn-G_12_, and dn-G_13_ did not affect propofol-induced dpLats1 and dpYAP. Interestingly, propofol-induced dpLats1 and dpYAP were abolished by the Rho inhibitor, C3 transferase, as well as by the Rho kinase (ROCK) inhibitor, Y27632, in HT-22 cells. We further determined which Rho kinases were involved. The results from cells transfected with different dn forms of Rho showed that RhoA was necessary for LPA-induced dpLats1 (Figure 5(c)).

### 3.6. No Interaction Was Observed between Calcineurin and YAP Activation in Hippocampal Neurons

**Figure 6:**
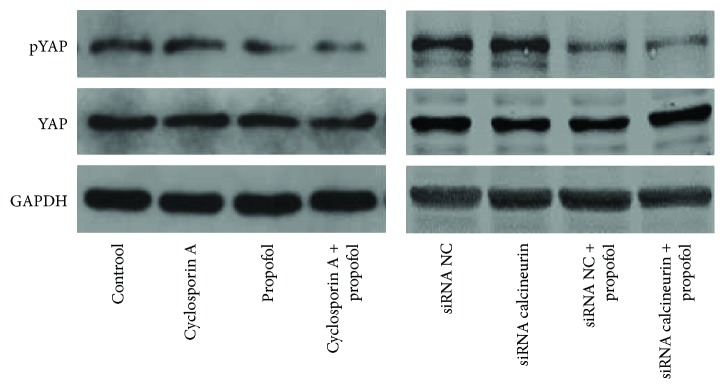
Calcineurin did not affect YAP activation in hippocampal neurons. HT-22 cells were pretreated with cyclosporin (1 *μ*mol/l for 2 hr) or transfected with different calcineurin shRNAs for 48 hr, then starved and treated with propofol (15 *μ*mol/l for 4 hr). Cell lysates were analyzed by Western blot to detect expression of YAP and pYAP.

Calcineurin is a member of the protein phosphatases (PP), also named protein phosphatase 2B (PP2B). Previous studies indicated that YAP activation could be mediated by PP, such as PP1 and PP2A [29, 30]. Thus, we aimed to clarify whether there is cross-talk between calcineurin and YAP activation in hippocampal neurons. As shown in Figure 6, inhibition of expression and activation of calcineurin did not affect YAP activation with or without propofol treatment, which indicated independent roles of calcineurin and YAP in propofol protection of hippocampal neurons from H/R-induced apoptosis.

## 4. Discussion

The hippocampus is functionally important in the central nervous system due to its vital role in memory and learning abilities of humans. Thus, hippocampal nerve cells damaged by I/R injury could lead to critical neurological dysfunction [31]. Recently, neuroprotective roles of propofol have been proved in an I/R model. However, fewer studies were designed to explore whether propofol could protect hippocampal neurons from I/R injury. In this study, a hypoxia-reoxygenation model stimulating I/R injury was used to treat hippocampal neurons. The results showed that treatment of propofol significantly suppressed apoptosis of hypoxia-reoxygenated hippocampal neurons.

Intracellular calcium overload is recognized as an initiator of cell apoptosis [26]. It is believed that I/R injury could induce the accumulation of intracellular calcium [32]. A recent study indicated that propofol could alleviate hippocampal neuronal injury induced by I/R through depressing calcium overload [6]. Consistently, our results showed a similar mechanism of propofol in protecting hippocampal neurons from I/R injury. The intracellular calcium concentration was regulated by calcium ion channels such as sarcoplasmic reticulum calcium ATPase (SERCA), ryanodine receptors (RyRs), and L-type calcium channel (LTCC). Calcineurin activity change was implicated in regulating the intracellular calcium by adjusting the opening of RyR via affecting FK506-binding protein 12.6 (FKBP12.6) [33, 34]. When encountering specific stimuli, the activity of calcineurin is upregulated to facilitate the splitting of the FKBP-RyR complex [35]. As a result, RyR channel opens to release calcium to induce calcium overload. In the present study, propofol was shown to decrease the enzymatic activity of calcineurin in hypoxia-reoxygenated hippocampal neurons, resulting in disassociating FKBP12.6 from RyR3 to close the channel, which reduced the intracellular calcium overload.

Propofol, as a widely used intravenous short-acting anesthetic, has been proved to regulate multiple intracellular signaling pathways [8]. Thus, we presented the hypothesis that other signaling pathways may also be involved in propofol's neuroprotective roles. Interestingly, we demonstrated that expression of YAP was crucial for propofol to protect hippocampal neuronal apoptosis from H/R injury. In addition, propofol could activate YAP by dephosphorylating YAP and promoting nuclear translocation. As a transcriptional coactivator, activated YAP can promote stem/progenitor cell self-renewal, drive cell migration and proliferation, and suppress cell apoptosis mainly through binding with TEAD in the nucleus [27]. Recent deep genome-wide sequencing led to multiple target genes of YAP. Among them, survival genes (e.g., survivin and Bcl2), proliferation-associated genes (e.g., Ctgf, Cyr61, c-Myc, Foxm1, and miR-130), differentiation-associated genes (e.g., Oct4, Nanog, Cdx2, and Pax3), and migration/invasion-associated genes (e.g., Ctgf, Cyr61, and Zeb2) were sorted [27]. Since propofol has been proved to promote hippocampal neuronal survival and inhibit apoptosis in our research, herein, we further found that propofol could induce expression of Bcl2 (rather than survivin), which is specifically considered as an important antiapoptotic protein.

YAP is a type of transcriptional coactivator with a PDZ-binding motif. Its dephosphorylated morphology could translocate into the nucleus to bind the TEAD transcription factor family and induce expression of a wide range of genes [27]. YAP activation was initially identified to be negatively regulated by Hippo pathway kinases via phosphorylation of Ser127, which results in YAP 14-3-3 binding, cytoplasmic retention, and degradation [36]. Previous studies showed that YAP phosphorylation could be mediated by cell contact, mechanical signals, stress signals, cell polarity/architecture, and cell cycle. However, the extracellular soluble regulators of YAP were barely known, until Yu et al. found bioactive lipids, LPA, and sphingosine-1-phosphate (S1P) as extracellular regulators of YAP in mammary cell lines [37]. In addition, several hormones have been proved to regulate YAP, such as epinephrine, estrogen, and glucagon [38]. In the present study, we demonstrated that propofol acted as another extracellular soluble regulator of YAP. Propofol could dephosphorylate Ser127 of YAP, which resulted in YAP activation and nuclear translocation. Mostly, soluble factors regulate YAP via the Hippo pathway by their cognate G-protein-coupled receptors and associated G-protein subunits to engage the small GTPases, RhoA, and ROCK, leading to alterations of activation of Lats1/2 [38]. Consistently, our data indicated propofol dephosphorylated Lats1/2 and YAP partly through RhoA and ROCK. However, the large G proteins, neither Gi, Gq, nor G_12/13_, were not involved, indicating that propofol acted independently of the G-protein-coupled receptor to regulate YAP activity. Thus, the details of propofol in regulation of small GTPases in hippocampal neurons need more exploration.

In the classical Hippo pathway, Lats1/2 is directly regulated by MST1/2. Recently, studies found that several phosphokinases and phosphatases were considered as upstream regulators of Lats1, which is parallel to MST1/2, such as PKA, NF2, MAP4K4, and AMPK. Interestingly, one study quantitatively analyzed global phosphoproteome alterations of HT-22 cells after propofol treatment revealed that propofol could mediate phosphorylation of NF2 and MAP4K4 [39]. Our data showed that propofol-induced dephosphorylation of Lats1/2 and YAP was partly absorbed by inhibition of RhoA. Thus, we speculated that propofol could also activate YAP through the NF2-Lats1/2 and MAP4K4-Lats1/2 pathways.

## 5. Conclusion

Our results indicate that propofol could protect hippocampal neurons from I/R injury through two independent signaling pathways, including the calcineurin/FKBP12.6-RyR/calcium overload pathway and the RhoA/Lats1/YAP/Bcl-2 pathway. This work further supports the potential therapeutic role of propofol against I/R in the nervous system.

## Data Availability

The data used to support the findings of this study are included within the article. Alternatively, the data are also available from the corresponding author upon request (caihui9@stu.xjtu.edu.cn).

## References

[1] Hankey G. J. (2017). Stroke. *The Lancet*.

[2] Sevick L. K., Ghali S., Hill M. D. (2017). Systematic review of the cost and cost-effectiveness of rapid endovascular therapy for acute ischemic stroke. *Stroke*.

[3] Han X. R., Wen X., Wang Y. J. (2018). Protective effects of microRNA-431 against cerebral ischemia-reperfusion injury in rats by targeting the Rho/Rho-kinase signaling pathway. *Journal of Cellular Physiology*.

[4] Yang Y., Li X., Zhang L., Liu L., Jing G., Cai H. (2015). Ginsenoside Rg1 suppressed inflammation and neuron apoptosis by activating PPAR*γ*/HO-1 in hippocampus in rat model of cerebral ischemia-reperfusion injury. *International Journal of Clinical and Experimental Pathology*.

[5] Lu Y., Chen W., Lin C. (2017). The protective effects of propofol against CoCl_2_-induced HT22 cell hypoxia injury via PP2A/CAMKII*α*/nNOS pathway. *BMC Anesthesiology*.

[6] Wang H., Zheng S., Liu M. (2016). The effect of propofol on mitochondrial fission during oxygen-glucose deprivation and reperfusion injury in rat hippocampal neurons. *PLoS One*.

[7] Tao T., Li C. L., Yang W. C. (2016). Protective effects of propofol against whole cerebral ischemia/reperfusion injury in rats through the inhibition of the apoptosis-inducing factor pathway. *Brain Research*.

[8] Vasileiou I., Xanthos T., Koudouna E. (2009). Propofol: a review of its non-anaesthetic effects. *European Journal of Pharmacology*.

[9] Zhang J., Xia Y., Xu Z., Deng X. (2016). Propofol suppressed hypoxia/reoxygenation-induced apoptosis in HBVSMC by regulation of the expression of Bcl-2, Bax, Caspase3, Kir6.1, and p-JNK. *Oxidative Medicine and Cellular Longevity*.

[10] Lemoine S., Zhu L., Gress S., Gérard J.-L., Allouche S., Hanouz J.-L. (2016). Mitochondrial involvement in propofol-induced cardioprotection: an in vitro study in human myocardium. *Experimental Biology and Medicine*.

[11] Noh H. S., Shin I. W., Ha J. H., Hah Y. S., Baek S. M., Kim D. R. (2010). Propofol protects the autophagic cell death induced by the ischemia/reperfusion injury in rats. *Molecules and Cells*.

[12] Zhao B., Li L., Lei Q., Guan K. L. (2010). The Hippo-YAP pathway in organ size control and tumorigenesis: an updated version. *Genes & Development*.

[13] Hoshino M., Qi M. L., Yoshimura N. (2006). Transcriptional repression induces a slowly progressive atypical neuronal death associated with changes of YAP isoforms and p73. *The Journal of Cell Biology*.

[14] Morimoto N., Nagai M., Miyazaki K. (2009). Progressive decrease in the level of YAPdeltaCs, prosurvival isoforms of YAP, in the spinal cord of transgenic mouse carrying a mutant SOD1 gene. *Journal of Neuroscience Research*.

[15] Cao X., Pfaff S. L., Gage F. H. (2008). YAP regulates neural progenitor cell number via the TEA domain transcription factor. *Genes & Development*.

[16] Lin Y. T., Ding J. Y., Li M. Y., Yeh T. S., Wang T. W., Yu J. Y. (2012). YAP regulates neuronal differentiation through Sonic hedgehog signaling pathway. *Experimental Cell Research*.

[17] Zhang H., Deo M., Thompson R. C., Uhler M. D., Turner D. L. (2012). Negative regulation of Yap during neuronal differentiation. *Developmental Biology*.

[18] Lavado A., He Y., Pare J. (2013). Tumor suppressor Nf2 limits expansion of the neural progenitor pool by inhibiting Yap/Taz transcriptional coactivators. *Development*.

[19] Moleirinho S., Patrick C., Tilston-Lünel A. M. (2013). Willin, an upstream component of the hippo signaling pathway, orchestrates mammalian peripheral nerve fibroblasts. *PLoS One*.

[20] Jia X. F., Ye F., Wang Y. B., Feng D. X. (2016). ROCK inhibition enhances neurite outgrowth in neural stem cells by upregulating YAP expression in vitro. *Neural Regeneration Research*.

[21] Yan F., Tan X., Wan W. (2017). ErbB4 protects against neuronal apoptosis via activation of YAP/PIK3CB signaling pathway in a rat model of subarachnoid hemorrhage. *Experimental Neurology*.

[22] Zhu C., Li L., Zhao B. (2015). The regulation and function of YAP transcription co-activator. *Acta Biochimica et Biophysica Sinica*.

[23] Santinon G., Pocaterra A., Dupont S. (2016). Control of YAP/TAZ activity by metabolic and nutrient-sensing pathways. *Trends in Cell Biology*.

[24] Chen X., Du Y. M., Xu F., Liu D., Wang Y. L. (2016). Propofol prevents hippocampal neuronal loss and memory impairment in cerebral ischemia injury through promoting PTEN degradation. *Journal of Molecular Neuroscience*.

[25] Chen M., Sun H. Y., Hu P. (2013). Activation of BKca channels mediates hippocampal neuronal death after reoxygenation and reperfusion. *Molecular Neurobiology*.

[26] Meng Y., Li W.-Z., Shi Y.-W., Zhou B.-F., Ma R., Li W.-P. (2016). Danshensu protects against ischemia/reperfusion injury and inhibits the apoptosis of H9c2 cells by reducing the calcium overload through the p-JNK-NF-*κ*B-TRPC6 pathway. *International Journal of Molecular Medicine*.

[27] Meng Z., Moroishi T., Guan K. L. (2016). Mechanisms of Hippo pathway regulation. *Genes & Development*.

[28] Zhang Z., Wang H., Jin Z. (2015). Downregulation of survivin regulates adult hippocampal neurogenesis and apoptosis, and inhibits spatial learning and memory following traumatic brain injury. *Neuroscience*.

[29] Zheng Y., Liu B., Wang L., Lei H., Pulgar Prieto K. D., Pan D. (2017). Homeostatic control of Hpo/MST kinase activity through autophosphorylation-dependent recruitment of the STRIPAK PP2A phosphatase complex. *Cell Reports*.

[30] Cai H., Xu Y. (2013). The role of LPA and YAP signaling in long-term migration of human ovarian cancer cells. *Cell Communication and Signaling: CCS*.

[31] Wei R., Zhang R., Xie Y., Shen L., Chen F. (2015). Hydrogen suppresses hypoxia/reoxygenation-induced cell death in hippocampal neurons through reducing oxidative stress. *Cellular Physiology and Biochemistry*.

[32] Vasques E. R., Cunha J. E. M., Coelho A. M. M. (2016). Trisulfate disaccharide decreases calcium overload and protects liver injury secondary to liver ischemia/reperfusion. *PLoS One*.

[33] Matsoukas M.-T., Aranguren-Ibáñez Á., Lozano T. (2015). Identification of small-molecule inhibitors of calcineurin-NFATc signaling that mimic the PxIxIT motif of calcineurin binding partners. *Science Signaling*.

[34] Liao B., Zheng Y. M., Yadav V. R., Korde A. S., Wang Y. X. (2011). Hypoxia induces intracellular Ca2+ release by causing reactive oxygen species-mediated dissociation of FK506-binding protein 12.6 from ryanodine receptor 2 in pulmonary artery myocytes. *Antioxidants & Redox Signaling*.

[35] Liu Z., Cai H., Zhu H. (2014). Protein kinase RNA-like endoplasmic reticulum kinase (PERK)/calcineurin signaling is a novel pathway regulating intracellular calcium accumulation which might be involved in ventricular arrhythmias in diabetic cardiomyopathy. *Cellular Signalling*.

[36] Wang K., Degerny C., Xu M., Yang X. J. (2009). YAP, TAZ, and Yorkie: a conserved family of signal-responsive transcriptional coregulators in animal development and human disease. *Biochemistry and Cell Biology*.

[37] Yu F. X., Zhao B., Panupinthu N. (2012). Regulation of the Hippo-YAP pathway by G-protein-coupled receptor signaling. *Cell*.

[38] Watt K. I., Harvey K. F., Gregorevic P. (2017). Regulation of tissue growth by the mammalian Hippo signaling pathway. *Frontiers in Physiology*.

[39] Zhang H., Ye J., Shi Z., Bu C., Bao F. (2017). Quantitative analyses of the global proteome and phosphoproteome reveal the different impacts of propofol and dexmedetomidine on HT22 cells. *Scientific Reports*.

